# Global burden of deaths from Epstein-Barr virus attributable malignancies 1990-2010

**DOI:** 10.1186/1750-9378-9-38

**Published:** 2014-11-17

**Authors:** Gulfaraz Khan, Muhammad Jawad Hashim

**Affiliations:** Department of Microbiology and Immunology, College of Medicine and Health Sciences, United Arab Emirates University, Al Ain, United Arab Emirates; Department of Family Medicine, College of Medicine and Health Sciences (Tawam Hospital Campus), United Arab Emirates University, Al Ain, P.O. Box 17666, United Arab Emirates

**Keywords:** EBV, Viral-associated cancers, Global cancer mortality, Cancer risk factors

## Abstract

**Background:**

Epstein-Barr virus (EBV) is an oncogenic virus implicated in the pathogenesis of a number of human malignancies of both lymphoid and epithelial origin. Thus, a comprehensive and up-to-date analysis focused on the global burden of EBV-attributable malignancies is of significant interest.

**Methods:**

Based on published studies, we estimated the proportion of Burkitt’s lymphoma (BL), Hodgkin’s lymphoma (HL), nasopharyngeal carcinoma NPC), gastric carcinoma (GC) and post-transplant lymphoproliferative disease (PTLD) attributable to EBV, taking into consideration age, sex and geographical variations. This proportion was then imputed into the Global Burden of Disease 2010 dataset to determine the global burden of each EBV-attributable malignancy in males and females in 20 different age groups and 21 world regions from 1990 to 2010.

**Results:**

The analysis showed that the combined global burden of deaths in 2010 from all EBV-attributable malignancies was 142,979, representing 1.8% of all cancer deaths. This burden has increased by 14.6% over a period of 20 years. All 5 EBV-attributable malignancies were more common in males in all geographical regions (ratio of 2.6:1). Gastric cancer and NPC accounted for 92% of all EBV-attributable cancer deaths. Almost 50% of EBV-attributed malignancies occurred in East Asia. This region also had the highest age-standardized death rates for both NPC and GC.

**Conclusions:**

Approximately 143,000 deaths in 2010 were attributed to EBV-associated malignancies. This figure is likely to be an underestimate since some of the less prevalent EBV-associated malignancies have not been included. Moreover, the global increase in population and life-expectancy will further increase the overall burden of EBV-associated cancer deaths. Development of a suitable vaccine could have a substantial impact on reducing this burden.

**Electronic supplementary material:**

The online version of this article (doi:10.1186/1750-9378-9-38) contains supplementary material, which is available to authorized users.

## Introduction

Cancer is one of the leading causes of death worldwide and research focused on understanding the etiology and pathogenesis of cancer is a major challenge. It has previously been estimated that oncogenic viruses play an etiological role in the development of approximately 12% of all human malignancies [1, 2]. The vast majority of these malignancies are caused by just five different viruses of which Epstein-Barr virus (EBV) is arguably one of the most extensively studied [3].

EBV is a large dsDNA lymphotropic herpesvirus historically associated with Burkitt’s lymphoma, from which the virus was first isolated 50 years ago [4]. However, ever since its isolation, EBV has continued to attract considerable attention, primarily due to its oncogenic properties and its association with a number of human malignancies, including Burkitt’s lymphoma (BL), nasopharyngeal carcinoma (NPC), post-transplant lymphoproliferative disease (PTLD), Hodgkin’s lymphoma (HL) and gastric carcinoma (GC) [3]. EBV is primarily transmitted via saliva and in healthy immunocompetent individuals it infects and establishes life-long latency in memory B-lymphocytes [5, 6]. In these cells, the virus limits its gene expression to 1 or 2 viral proteins only, thus escaping the immune surveillance [7]. This is referred to as type 0/1 latency. In some EBV-associated malignancies, such as NPC and HL, at least 3 viral genes have been shown to be expressed, including the oncogenic membrane protein LMP-1 [3]. This is referred to as type 2 latency. How the viral infected cells in these malignancies escape the immune system is unclear. In contrast, *in vitro* infection of B-lymphocytes results in their immortalization and establishment of lymphoblastoid cell lines (LCLs) [8]. In these cells, at least a dozen different EBV latent products are expressed, including 6 Epstein-Barr nuclear antigens (EBNAs), 3 latent membrane proteins (LMPs) and 2 non-protein coding small RNAs (EBERs) [3]. In addition to these, a number of micro-RNAs have also been shown to be expressed [9]. This is referred to as type 3 latency or growth program [10, 11]. A substantial body of evidence indicates that a number of these latent viral products are central for EBV-induced immortalization of the infected cells. In EBV-associated malignancies, all three patterns of latency have been detected, suggesting that EBV induces oncogenesis by different mechanisms in different malignancies. Although EBV is carried asymptomatically by over 90% of adults worldwide, induction of cancer by this virus is nevertheless a very rare event. This clearly indicates that EBV on its own is not sufficient and other co-factors are necessary [12–14]. Thus, in order to link EBV in the etiology of a malignancy it is essential to demonstrate the presence of viral genome and/or gene expression directly in the tumor cells. This approach has revealed that the virus is not necessarily etiologically involved in all cases of a malignancy with which EBV has been implicated [15, 16]. For example, only about 40% of Hodgkin’s lymphoma cases have EBV in the malignant cells and the prevalence varies with age [17].

In this study we provide the most up-to-date and detailed descriptive epidemiology of EBV-attributable malignancies using the Global Burden of Disease, Injuries and Risk Factors Study 2010 (GBD 2010) dataset. GBD 2010 is the largest and most comprehensive study ever conducted to measure the global health metrics [18, 19]. As far as we are aware, the present study is the first to use GBD 2010 data to estimate the burden of EBV-associated malignancies in different age and sex groups in 21 world geographical regions from 1990 to 2010. It is hoped that the findings of this study will high-light the need for potential preventative measures and the global regions where implementation of such measures would have the greatest impact.

## Methods

### EBV associated malignancies

EBV is an accepted carcinogen [20] and experimental studies have clearly demonstrated that it is present in the tumor cells of several malignancies, including NPC, BL, HL, GC and PTLD [3, 21]. There is substantial evidence that EBV has a causative role in the pathogenesis of these malignancies. However, the association is not universal and not all cases from all regions are linked to EBV. Our first step in this study was to estimate the proportion of these malignancies that can be reliably attributed to EBV based on published studies.

### Estimation of the proportion of EBV-attributable cases

Since EBV is ubiquitous in the general population, EBV-attributable cancers are defined as those in which viral DNA/RNA and/or viral gene expression can be demonstrated in the tumor tissues. Based on published studies, we estimated the proportion of NPC [1, 2], GC [22, 23], HL [17, 24–29], BL [1, 2] and PTLD [30, 31] that are attributable to EBV, taking into consideration any established variations that have been reported in different age, sex and ethnic groups (Table 1). For GC, the proportion of EBV-attributable cases has been reported to be similar in different world regions [22, 32], but varies significantly with gender [22, 33, 34]. Based on two large meta-analysis studies, we have used EBV-attributable estimates of 11% and 6% for males and females respectively [22, 23]. For HL, there is a general consensus that the EBV-attributable fraction varies significantly between different age groups. From the published studies, we estimated 62%, 30% and 55% of the cases to be attributed to EBV for the age groups 0-14 years [17, 24, 27, 29], 15-54 years [17, 25, 27–29], and 55-80+ years [17, 26–29] respectively. For PTLD, we estimated that 80% of PTLD cases to be attributed to EBV [30, 31] (Table 1).

**Table 1:** **EBV-associated malignancies**

Malignancy	Type of EBV latency	Prevalence of EBV in cases (%)	Comment
**Nasopharyngeal carcinoma (ICD10:C11)**	Type II		Estimates based on 2 previous studies on global burden of infection-associated cancer [1, 2].
• High/intermediate incidence region		100%	
• Low incidence region		80%	
			High/intermediate regions: East Asia, South Asia, South East Asia & North Africa & Middle East [53]. All other regions were regarded as low incidence.
**Gastric carcinoma (ICD10:C16)**	Type II		Estimate based on 2 large meta-analysis studies with a cumulative total of 25,690 cases [22, 23].
• Males		11%	
• Females		6%	
**Hodgkin’s disease (ICD10:C81)**	Type II		Estimates based on 7 studies with cumulative total of 3357 cases [17, 24–29].
• Children <14yrs		62%	
• Adults 15-54yrs		30%	Age group 0-14 yrs based on 4 studies [17, 24, 27, 29].
• Adults >55yrs		55%	
			Age group 15-54 yrs based on 5 studies [17, 25, 27–29].
			Age group 55+ yrs based on 5 studies [17, 26–29].
**Burkitt’s Lymphoma (ICD10:C83.5)**	Type I		Estimates based on 2 previous studies on global burden of infection-associated cancer [1, 2].Endemic region: Sub-Saharan Africa.
• **Endemic**			
90.5% of all NHL are BL in 0-14 age group [1].		95%	
• **Intermediate**			Intermediate regions: N. Africa & Middle east, Latin America.
33.3% of all NHL are BL in 0-14 age group [1].		50%	
• **Non-endemic**			Non-endemic: All other regions.
15.2% of all NHL are BL in 0-14 age group [1].		20%	BL is 3-4x more common in males [41–43, 45]. In this study we have used male:female ratio of 3:1.
In adults (age group 15-80+), irrespective of region, BL is estimated to constitute 2% of all NHL [39].			
**Post-transplant lymphoproliferative disease (ICD10:D47.Z1)**	Type III		80% of all PTLD were estimated to be due to EBV [30, 31]. It was assumed the risk of developing PTLD and dying from it was the same for both sexes.
• 1.5% of transplant recipients were estimated to developed PTLD and 50% of these died with first year of post-transplant [30, 48, 49].		80%	

### Estimation of the mortality from NPC, GC, HL, BL and PTLD

Data files for mortality estimates of all cancer cases were obtained from the Institute of Health Metrics Evaluation (IHME), University of Washington [35]. Detailed descriptions of how mortality figures were estimated has been previously published as part of the GBD 2010 study [36, 37]. Briefly, mortality estimates were based on several different sources, including surveys, censuses, sample registration data and vital registration data, and final estimates derived using a range of statistical models [36–38]. All mortality figures and rates were estimated with 95% confidence intervals.

Age and sex-specific mortality estimates in 21 geographical regions were directly available for NPC, GC and HL from the GBD 2010 dataset. For BL, mortality data was not directly available as this malignancy was part of a broader category of non-Hodgkin’s lymphomas (NHL). Based on a previous study on the global burden of infection-associated cancers [1], the proportion of BL within the larger NHL category in the age group 0-14 years was estimated to be 90.5%, 33.3% and 15.2% for regions where BL is endemic, intermediate or sporadic respectively (Table 1). For age group 15-80+, irrespective of geographical region, the proportion of BL in HIV-negative adults was conservatively estimated to be 2% of all NHL [39]. BL is approximately 3-4 times more common in males as compared to females [40–45]. In this study we used male:female ratio of 3:1 in calculating the prevalence of BL. Thus, all proportions were stratified by age, sex and region. For estimating the prevalence of PTLD, we used data from Global Observatory on Donation and Transplantation (GODT), produced by the WHO-ONT collaboration [46]. In 2010, a total of 101,990 transplants (kidney, heart, liver, lung and pancreas) were performed, approximately 60% of which were on males [47]. Based on previous reports [30, 48], we estimated that approximately 1.5% of transplant recipients develop PTLD and 50% of these die within the first year of lymphoma development [30, 48, 49]. It was assumed that the risk of developing PTLD and dying from it was the same for both sexes.

### Estimation of the mortality from EBV-attributed NPC, GC, HL, BL and PTLD

The estimates of the proportion of EBV-attributable death for each malignancy established from the published literature (Table 1) were imputed into GBD 2010 data, adjusted for age, sex and geographical region. For example, for BL in East Sub-Saharan Africa, GBD 2010 dataset shows 227 deaths from NHL in males aged 1-4 years. In this region, 90.5% of NHL have been estimated to be BL cases in this age group [1], with a male predominance of 3:1 [41–45]. Based on this, 227 × 0.905 × 0.75 gives an estimated number of BL cases to be 154. Since 95% of BL cases in this age group and region are associated with EBV [1, 2], the number of EBV-attributed BL deaths can be estimated to be 146 cases. Using this approach, we calculated the burden of death from each EBV-associated malignancy in males and females in 20 different age groups and 21 different geographical regions for the year 2010. This was then extended for 5 different time points from 1990 to 2010.

## Results

### Overall global burden of EBV-attributed malignancies

Over a period of 20 years, global mortality from cancer has increased from 5.779 million in 1990 to 7.978 million in 2010. This is an increase of approximately 2% per year. However, the collective global number of deaths from NPC, BL, HL, GC and PTLD has remained fairly constant (a modest increase of only 0.2%). Of the total of 842,674 deaths from these 5 malignancies in 2010, 142,979 (17.0%) were calculated to be from EBV-attributed cases (Table 2). This represents 1.8% of all cancer deaths in 2010 worldwide. The largest number of deaths from EBV-attributed malignancies was for gastric carcinoma (69,081 cases), closely followed by NPC (63,118 cases). The proportion of cases of NPC, BL, HL, GC and PTLD, specifically adjusted for age, sex and geographical region, attributable to EBV was 97.2%, 51.2%, 44.7%, 9.2% and 80% respectively (Table 2).

**Table 2:** **Global burden of deaths from EBV-attributed malignancies in 2010**

Type of malignancy	Global deaths: All cases			Global deaths: EBV-attributed cases			% deaths from EBV-attributed cases (both)
	Males	Females	Both	Males	Females	Both	
NPC	45,640	19,264	64,904	44,418	18,700	63,118	97.2
BL	3,575	821	4,396	1,820	431	2,251	51.2
HL	10,208	7,510	17,718	4,507	3,410	7,917	44.7
Stomach	475,759	279,132	754,891	52,333	16,748	69,081	9.2
PTLD	459	306	765	367	245	612	80.0
Total	535,641	307,033	842,674	103,445	39,534	142,979	17.0

### Patterns of EBV-attributed malignancies by geographical region and time

**Figure 1:**
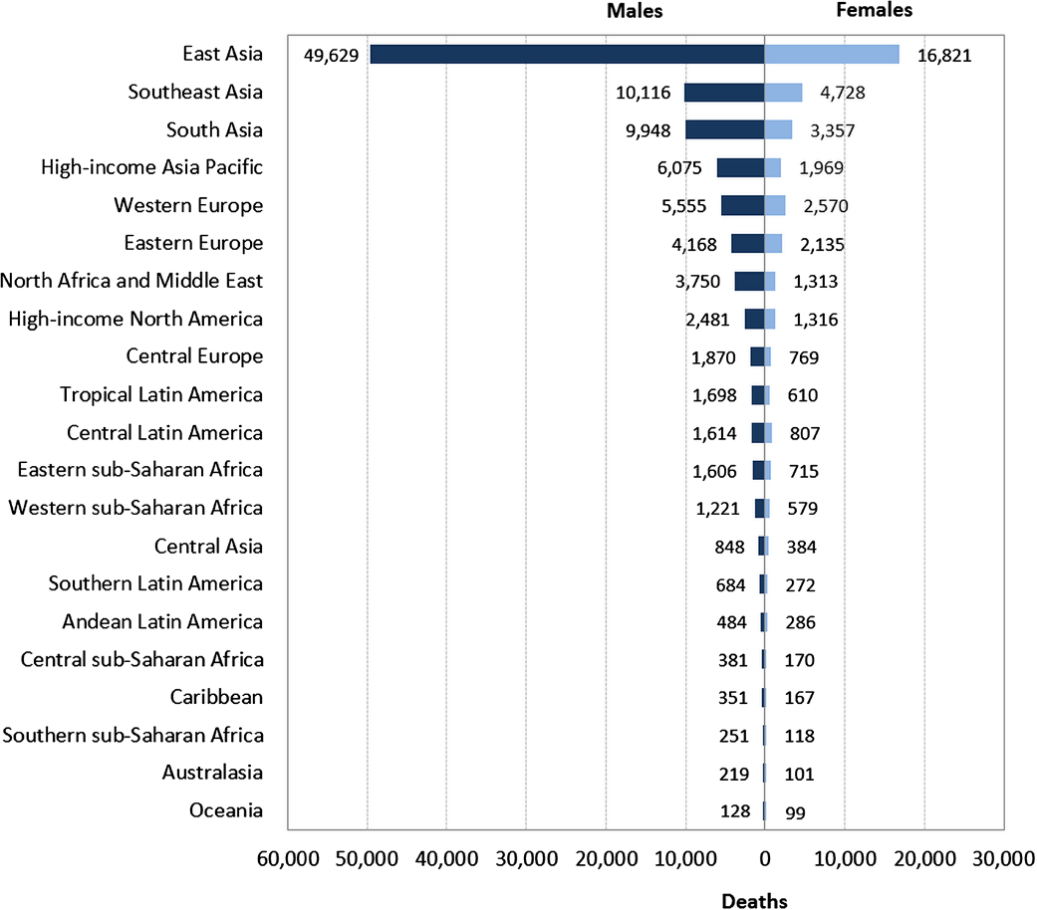
**Global burden of deaths from all EBV-attributed malignancies in 2010 by world regions.**

**Figure 2:**
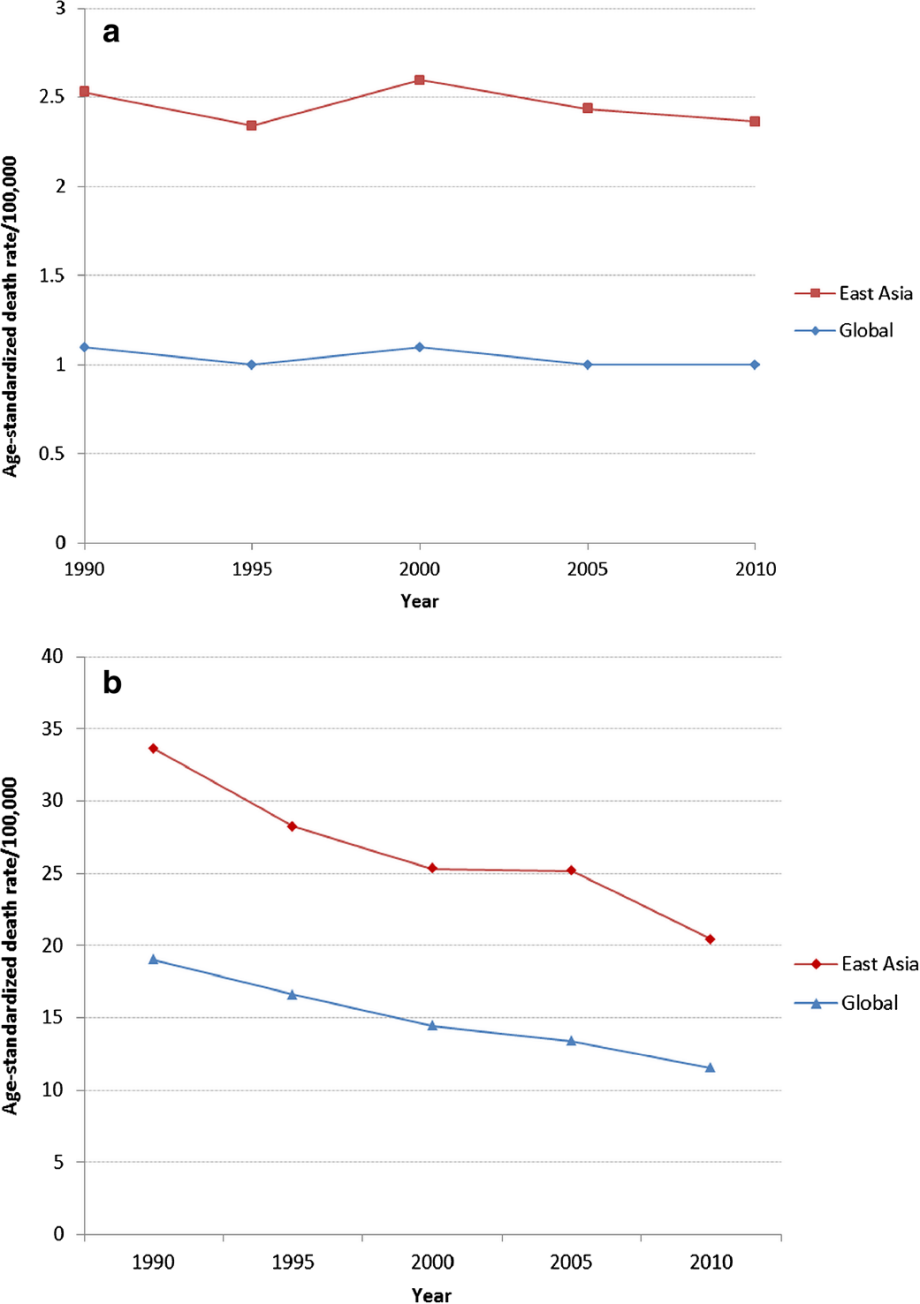
**Global burden of age standardized death rates of (a) nasopharyngeal carcinoma and (b) gastric cancer, 1990-2010.**

**Figure 3:**
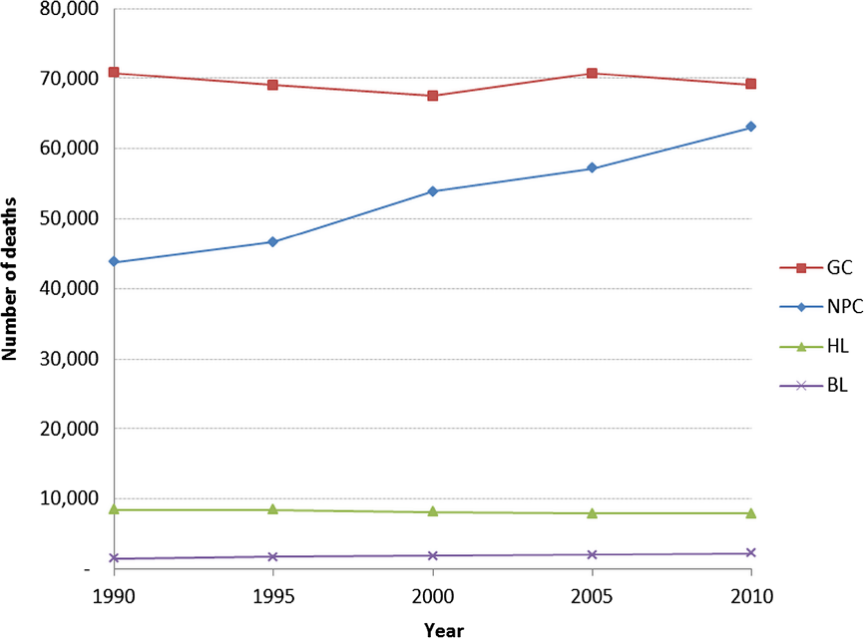
**Global burden of deaths from EBV-attributed malignancies, 1990-2010.**

Analysis of EBV-attributed malignancies in 21 world regions revealed that the highest mortality was in East Asia (Figure 1). In fact 47% of all EBV-attributed malignancies occurred in this region. This in turn is a reflection of the fact that this region, which includes China, Democratic People’s Republic of Korea and Taiwan, has by far the highest prevalence of both gastric and nasopharyngeal carcinoma in the world (Additional file 1: Figure S1). Age-standardized mortality rates for these malignancies in East Asia are also the highest in the world (Figure 2a and b). Furthermore, unlike the other malignancies, the burden of mortality due to NPC has increased from 43,828 in 1990, to 63,118 in 2010, an average annual increase of 2.2% (Figure 3). Although the age-adjusted death rate of NPC in East Asia is by far the highest in the world (2.5/100,000 in 2010), the rates have not increased over the 20 years (Figure 2a). This indicates that the increase in burden of NPC observed over the 20 years is most likely due to an increase in the population at risk.

### Patterns of EBV-attributed malignancies by sex and age

**Figure 4:**
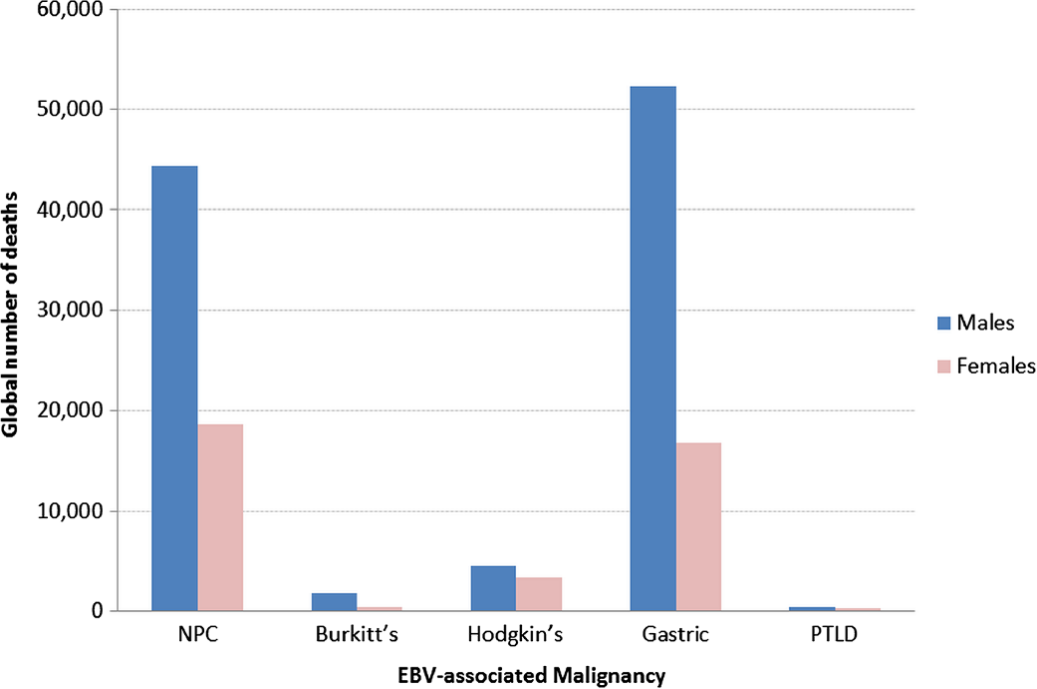
**Global burden of deaths from EBV-attributed malignancies in 2010.**

**Figure 5:**
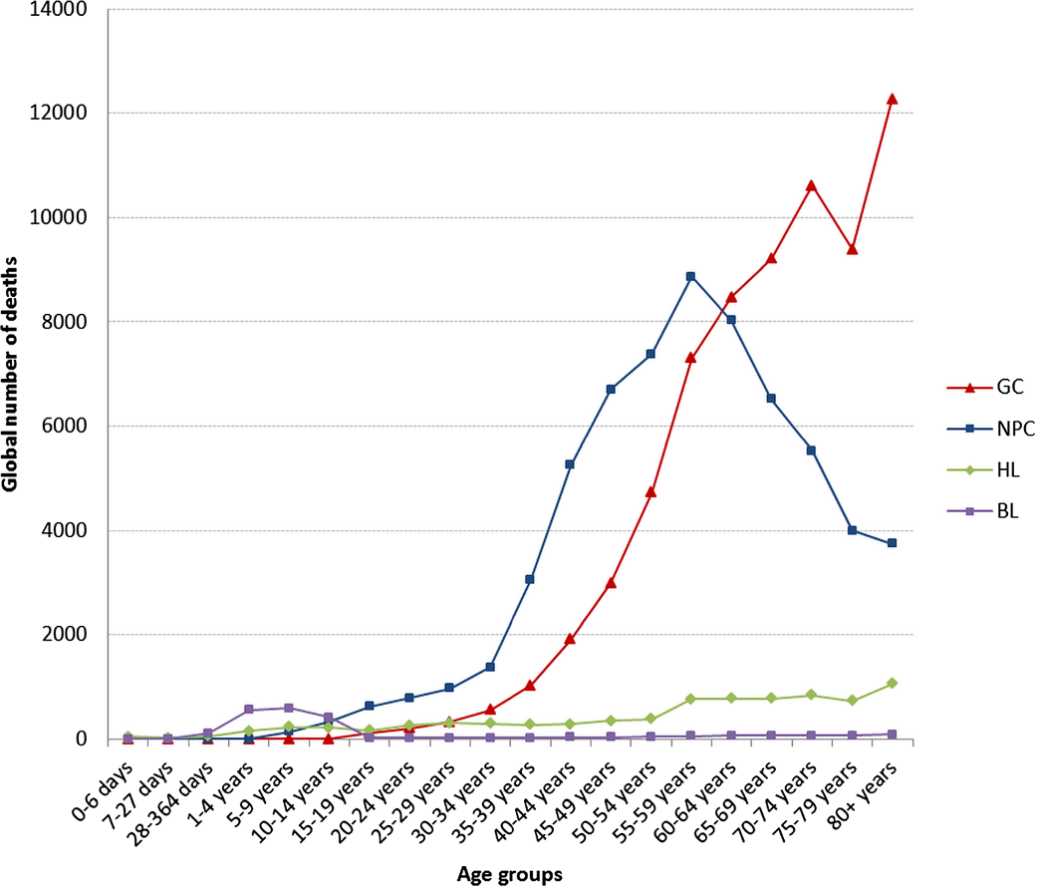
**Global burden of deaths from EBV-attributed malignancies by age in 2010.**

Global deaths from EBV-attributed cases of all 5 malignancies were up to 2.6 times higher in males as compared to females (Figure 4). This difference is likely to be an underestimate, since we did not take into account the accumulating data which indicates that males are more likely than females to have EBV-attributable HL [29, 50–52]. Furthermore, this male predominance was common in virtually all world regions (Additional file 1: Figure S1). The reason for this male preponderance is not known, but male genetics and male lifestyle are plausible risk factors. Analysis of deaths from these malignancies by age revealed that the vast majority of the cases occurred in adults, primarily after the age of 35 years (Figure 5). An exception to this was BL, which as expected, peaked in children between the ages of 1-5 years. Interestingly, the prevalence of NPC, unlike GC and HL did not continue to increase with age. Rather, it peaked in the age group 55-60 year-olds and thereafter decreased at an average of 2.75% annually (Figure 5). This trend is consistent with previous reports [53, 54].

## Discussion

Epstein-Barr virus is a well-recognized carcinogen implicated in the etiology of several malignancies of both epithelial and lymphoid origin. In this study, we present descriptive epidemiology of EBV-attributable malignancies using the GBD 2010 data. In contrast to previous studies [1, 2, 55], we focused exclusively on EBV-attributable cancers with an aim to provide an in-depth analysis of the malignancies associated with this virus. In addition to NPC, BL and HL, the current report also includes mortalities from GC and PTLD, both of which are known to be associated with EBV [42, 56, 57]. In this analysis, we present the global burden of mortality from EBV-attributed malignancies, stratified by age, sex and geographical region from 1990-2010. The results of this study demonstrate that the global burden of mortality from EBV-attributed malignancies accounts for 1.8% of all cancer deaths in 2010. This is a 14.6% increase from 1990 and the trends indicate that this burden will continue to increase as the world population and life-expectancy increase [37]. Gastric cancer and NPC accounted for 92% (132,199 cases) of all EBV-attributed cancer deaths, with the vast majority occurring in developing countries, in particular East Asia. Indeed, the age-standardized rates of both of these malignancies are also considerably higher in East Asia compared to western countries, consistent with previous reports [58, 59]. The reason for this elevated incidence in certain Asian countries remains unknown, as does the male preponderance [58]. Epidemiological studies on NPC and GC have shown that individuals who migrate from high-risk countries to low-risk countries have incidence rates intermediate to their country of origin and their host country [53, 59]. This implies that the etiology of these malignancies is complex and most probably involves multiple factors including, environmental, genetic and dietary. One factor in particular, namely EBV, has been consistently shown to be involved in the development of these malignancies [53, 56, 60], but the molecular mechanism(s) involved is not well understood. The fact that virtually all adults worldwide are infected with EBV, and yet only a very small fraction of individuals actually develop these malignancies, clearly indicates that EBV alone is not sufficient. For NPC, it has been hypothesized that infection with EBV early in childhood, which is typical of high-incidence regions, is important [53]. For GC, in particular non-cardia type, *Helicobacter pylori* is generally accepted to be one of the prime risk factors [61, 62]. Of the dietary and life style factors, increased intake of salts or salt preserved food, alcohol and smoking have been implicated, although the attributable risk is at best only modest [53, 63, 64].

In contrast to NPC and GC, the role of EBV in the development of BL, PTLD and HL is to some extent better understood. Burkitt’s lymphoma is primarily a childhood malignancy endemic in Sub-Saharan Africa. Three factors have been shown to be important in the development of this malignancy: EBV, malaria and deregulated activation of the c-*myc* oncogene [65]. In the case of PTLD, EBV is thought to be the primary driving force. EBV infected cells express several viral latent products [66, 67], including the viral oncogene LMP-1 [68]. These cells would normally be eliminated by the immune system, but in immunocompromised individuals such as transplant recipients, the infected cells proliferate unchecked. Reversal of immunosuppression or infusion of EBV-specific cytotoxic T-cells can prevent the development of PTLD [69, 70]. In HL, there is restricted EBV-gene expression in the malignant cells, but crucially LMP-1 is expressed [71] and thought to be central in the oncogenic process [72].

Although this study presents the most comprehensive and most up-to-date estimates of the global mortalities from EBV-associated malignancies, it has several limitations inherent in any study of this kind. First, our estimates rely on the accuracy of the dataset from the GBD 2010 study. GBD 2010 is the largest and most comprehensive project ever conducted to measure global health metrics and as expected, this ‘super-human’ effort had its own limitations which have been described in detail elsewhere [19, 37, 38]. Second, in calculating the mortality of EBV-attributable fraction of NPC, GC, HL, BL and PTLD, it was assumed that the risk of death from EBV-positive and negative cases is the same. This may not always be the case for all EBV-associated malignancies [25, 28, 73]. Indeed, some studies have reported a better prognosis for EBV-positive cases compared to negative cases [25, 74]. Third, to calculate the mortality of EBV-attributable cases of BL, we first had to determine the number of deaths from BL, as this was not directly available from GBD 2010 data. In the GBD 2010 data, BL was grouped in the larger category of non-Hodgkin’s lymphoma (NHL). In calculating the mortality of BL, it was assumed that the mortality of BL was the same as other lymphomas in the NHL group. Once again, this assumption is strictly speaking not true, since NHL represent a heterogeneous group of lymphomas with differing prognoses [75]. Fifth, for calculating the proportion of EBV-attributable malignancies at different time points i.e. 1990, 1995, 2000 and 2005, we used EBV-attributable proportions of 97.2% for NPC, 80% for PTLD, 51.2% for BL, 44.7% for HL and 9.2% for GC, estimated for 2010, with the assumption that these proportions have not changed over time. Finally, our estimate of 142,979 global deaths from EBV-associated malignancies is likely to be an underestimate since a few other EBV-associated malignancies such as central nervous system malignancies occurring in AIDS patients, for which there is substantial evidence for causality [76] have not been considered in this analysis.

## Conclusion

Cancer is amongst the leading causes of death. In 2010, cancer accounted for 7.978 million deaths, and this figure appears to be rising at a rate of approximately 2% per year [38]. Thus, understanding the risk factors or causes of cancer is of paramount importance for any future prevention strategies. The analysis presented here indicates that 1.8% of all cancer deaths in 2010 were associated with EBV. This is a sizable number of deaths and developing an effective vaccine would not only reduce this burden, but could also prevent infectious mononucleosis, which is also known to be caused by EBV [77].

## Electronic supplementary material

Additional file 1: Figure S1: Global burden of death from EBV-attributed malignancies in 2010 by region. (A) Gastric cancer (B) Nasopharyngeal carcinoma (C) Hodgkin’s lymphoma (D) Burkitt’s lymphoma. (PDF 210 KB)
