# Supplementary material for: Global burden of deaths from Epstein-Barr virus attributable malignancies 1990-2010
Source: Infect Agent Cancer. 2014 Nov 17;9:38. doi: 10.1186/1750-9378-9-38 (PMC4253616; doi:10.1186/1750-9378-9-38)

SUPPLEMENTARY INFORMATION

Supplementary Figure S1 - Global burden of death from EBV-attributed malignancies in 2010 by region

(A) Gastric cancer

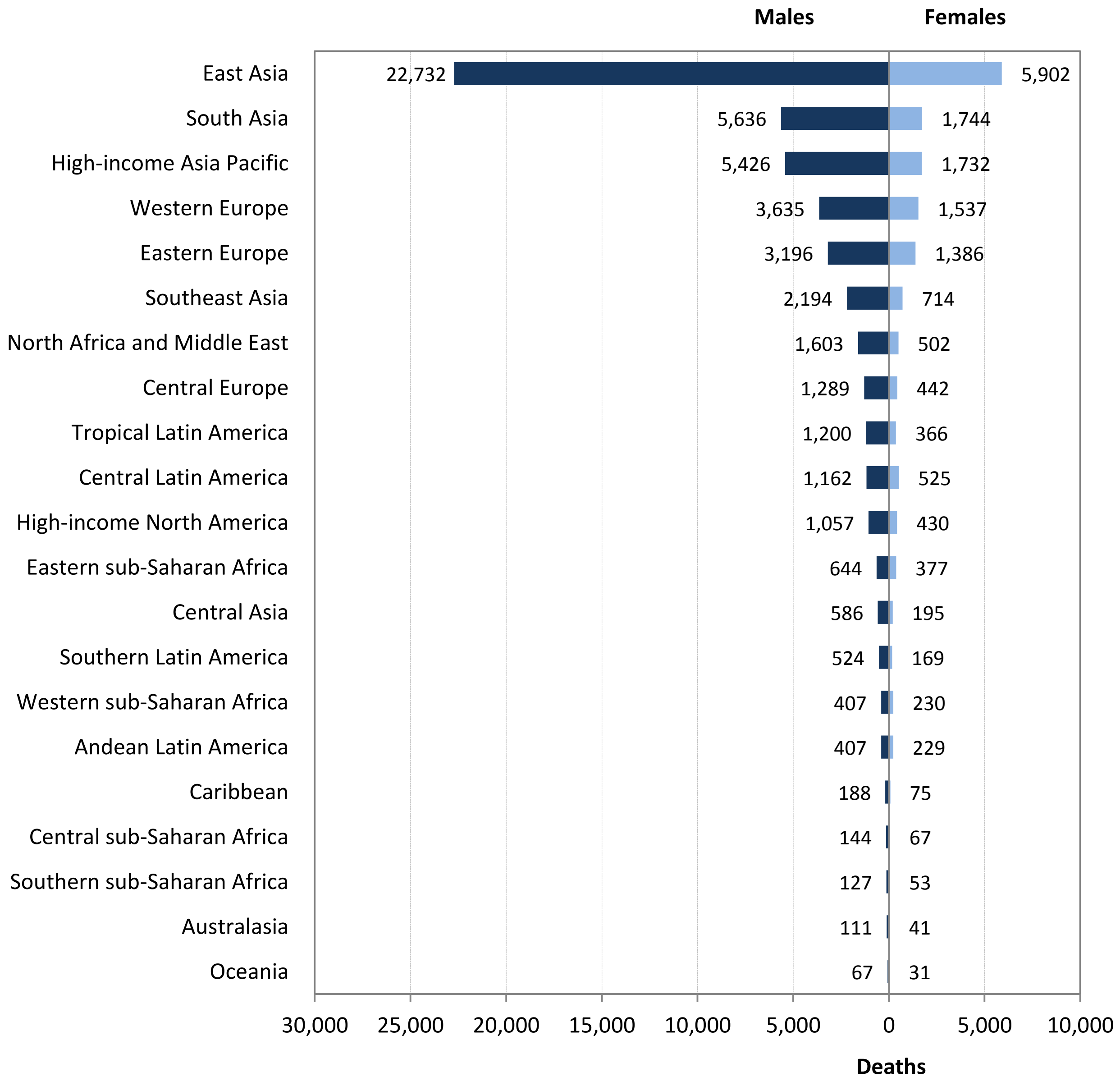

(B) Nasopharyngeal carcinoma

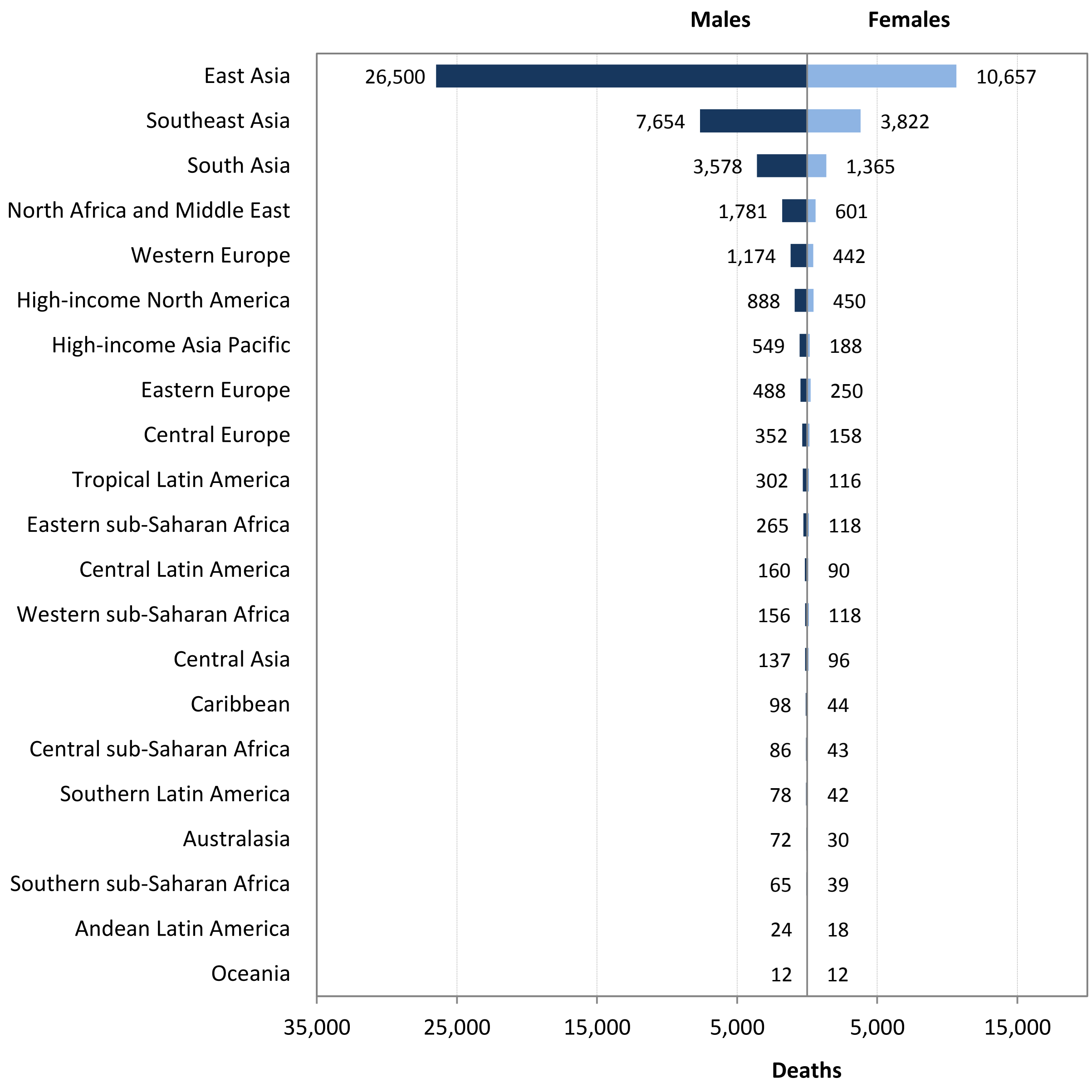

(C) Hodgkin's lymphoma

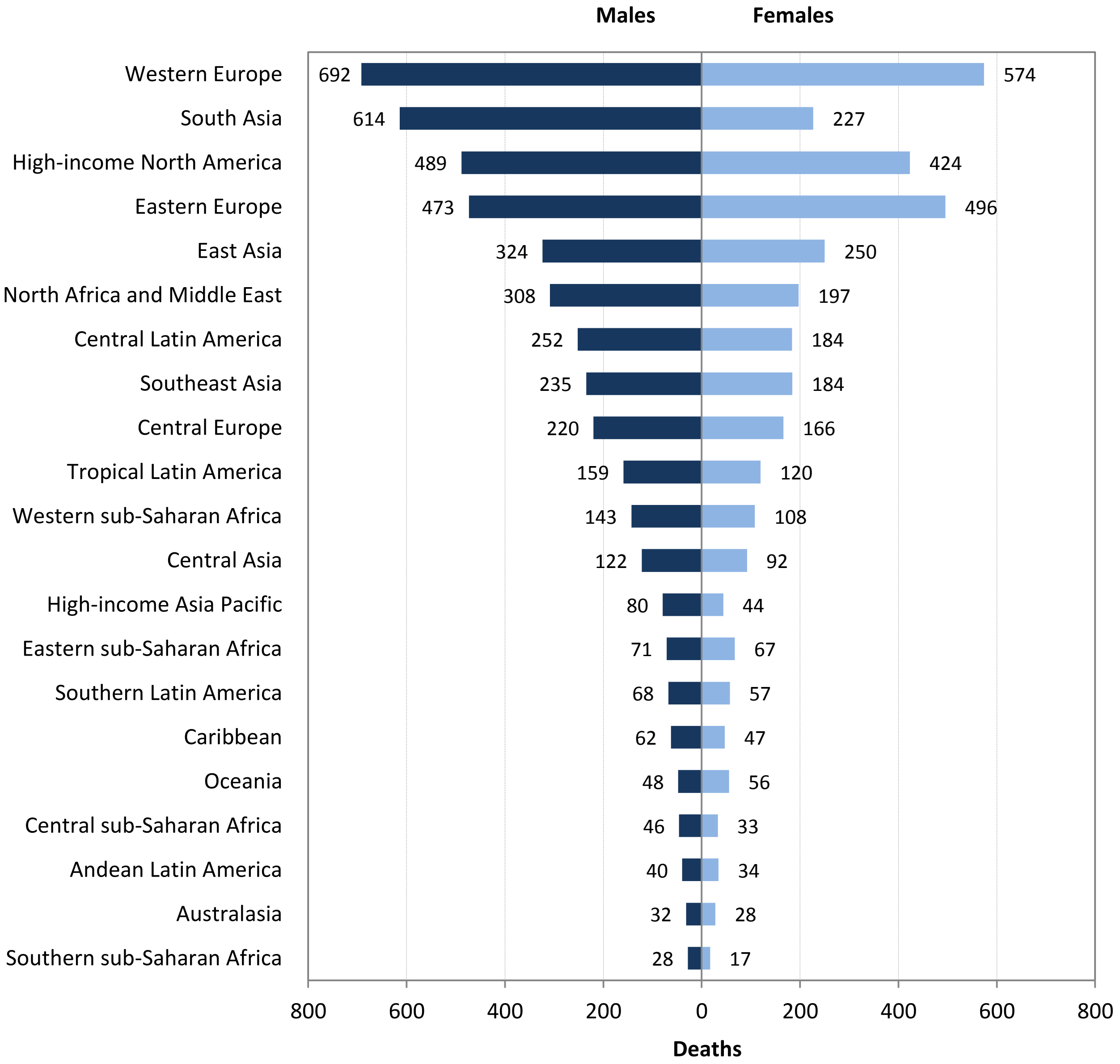

(D) Burkitt's lymphoma

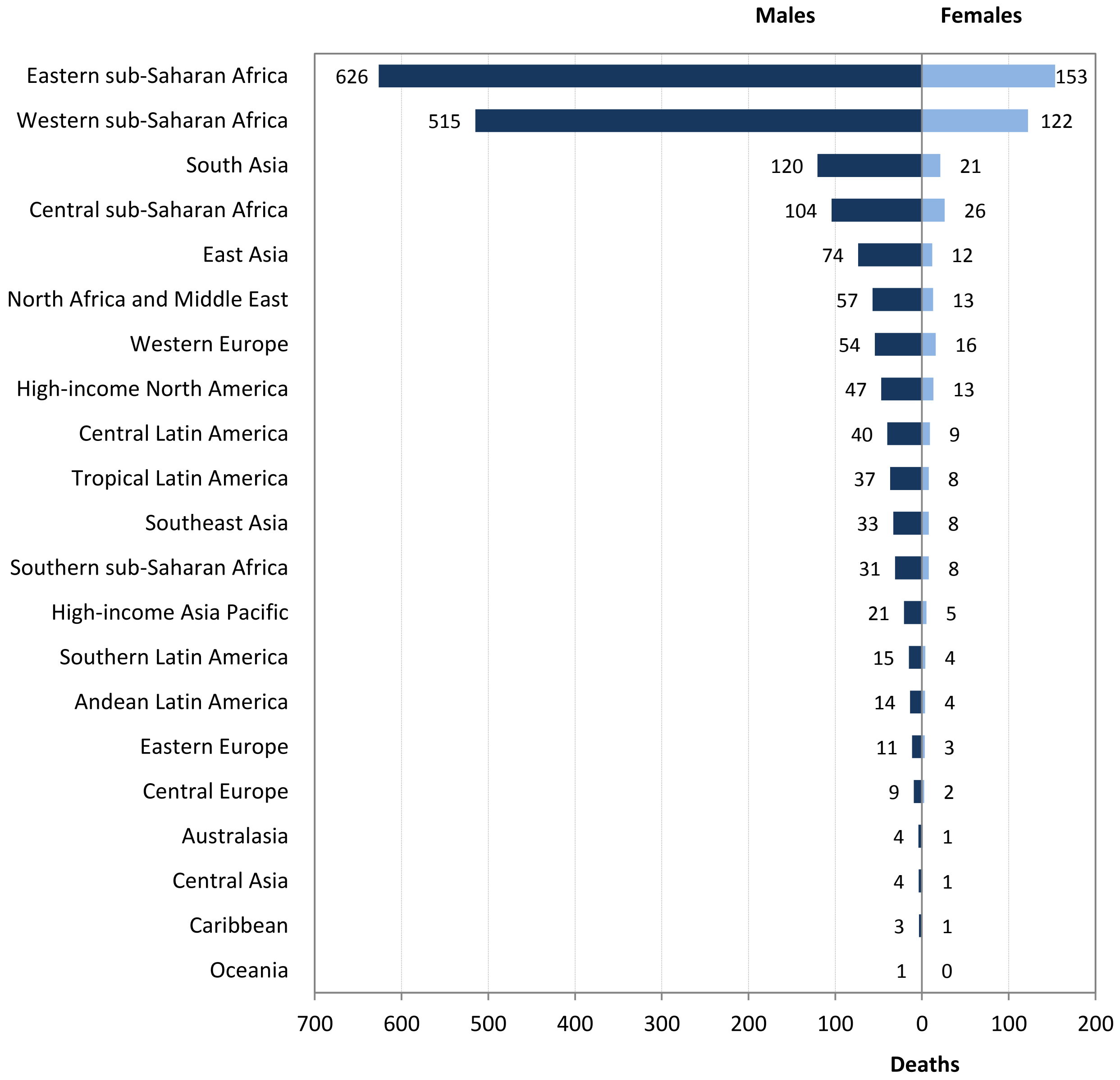

Supplement: Supplementary file 1 — Additional file 1: Figure S1: Global burden of death from EBV-attributed malignancies in 2010 by region. (A) Gastric cancer (B) Nasopharyngeal carcinoma (C) Hodgkin’s lymphoma (D) Burkitt’s lymphoma. (PDF 210 KB) [file 13027_2014_508_MOESM1_ESM.pdf]
